# The trinity of T cell engagement: navigating the molecular and clinical landscape of CAR-T, TILs, and TCEs in the war against cancer

**DOI:** 10.3389/fimmu.2026.1847986

**Published:** 2026-06-01

**Authors:** Qiang Yang, Shaobin Wang, Fanlin Liu, Yongli Yu, Hai Zhao

**Affiliations:** 1 Department of Neurosurgery, Lanzhou University Second Hospital, Lanzhou, Gansu, China; 2 Department of Operation Room, Lanzhou University Second Hospital, Lanzhou, Gansu, China; 3 Department of Neurosurgery, The Affiliated Hospital of Qingdao University, Qingdao, Shandong, China

**Keywords:** adoptive cell therapy, antigen escape, bispecific T cell engagers, CAR T-cells, immunosuppression, synthetic biology, tumor microenvironment, tumor-infiltrating lymphocytes (TILs)

## Abstract

The advent of cancer immunotherapy has fundamentally restructured the oncological paradigm, moving away from agents that directly target tumor cell kinetics toward strategies that empower the host immune system to recognize and eliminate malignancy. Central to this revolution is the cytotoxic T lymphocyte (CTL), now harnessed as a potent “living drug” through engineered and naturally selected modalities. This review provides a critical, in-depth examination of the three dominant pillars of T cell-driven therapies: Chimeric Antigen Receptor T-cell (CAR-T) therapy, Tumor-Infiltrating Lymphocyte (TIL) therapy, and T Cell Engagers (TCEs). We dismantle the molecular mechanisms defining each approach, contrasting the synthetic, major histocompatibility complex (MHC)-independent signaling of CAR-T cells with the diverse, MHC-restricted TCR repertoire of TILs, and the transient, pharmacologic bridging provided by bispecific TCEs. While CAR-T therapy has achieved historic success in hematologic malignancies, its translation to solid tumors is severely compromised by the hostile tumor microenvironment (TME), characterized by metabolic insulation, physical exclusion, and profound immunosuppression. Conversely, TIL therapy offers a polyclonal strategy tailored for solid tumors but is hindered by complex biomanufacturing logistics and variable tumor immunogenicity. TCEs promise off-the-shelf accessibility but face challenges regarding persistence and on-target/off-tumor toxicity. Beyond clinical outcomes, we explore the pathophysiological underpinnings of resistance, including antigen escape mechanisms and T cell exhaustion programs. Finally, we posit that the future of curative regimens lies in rational combinatorial strategies—integrating advanced genetic engineering, metabolic reprogramming, and TME-modulating agents like oncolytic viruses—to overcome the multifaceted defenses of solid tumors.

## Introduction

The historical trajectory of cancer treatment has long been dominated by therapies targeting the intrinsic vulnerabilities of rapidly dividing cells—namely, surgery, chemotherapy, and radiation (1). While these modalities remain foundational, their efficacy is often capped by a lack of specificity, resulting in significant systemic toxicity and an inability to eradicate dormant micro metastatic disease. The conceptual shift toward immunotherapy, rooted in *William Coley’s* early observations and solidified by the modern understanding of immune surveillance, posits that the immune system, particularly the adaptive branch, possesses the requisite specificity, potency, and memory to achieve durable cancer control (2).

The central effector in the antitumor response is the CD8+ cytotoxic T lymphocyte. However, the spontaneous generation of effective antitumor T cell responses is frequently thwarted by the tumor’s evolution under Darwinian immune pressure. Tumors exploit mechanisms of peripheral tolerance, downregulate antigen presentation machinery, and cultivate an immunosuppressive microenvironment to evade destruction (3–5). The modern era of clinical immunotherapy is defined by strategies designed to overcome these evasion tactics, either by releasing preexisting brakes on T cells or by directly supplying large numbers of effector T cells (6–9).

This review focuses on the latter categories, specifically analyzing the three most prominent T cell-centric modalities that have entered clinical practice: Chimeric Antigen Receptor T-cell (CAR-T) therapy, Tumor-Infiltrating Lymphocyte (TIL) therapy, and T Cell Engagers (TCEs). Each represents a distinct philosophical and engineering approach to the same problem: ensuring a potent cytotoxic T cell meets its cognate tumor antigen. CAR-T cells represent the triumph of synthetic biology, genetically reprogramming T cells to see surface targets independently of MHC presentation. TIL therapy leverages nature’s own selection process, expanding the polyclonal repertoire of T cells that have already successfully infiltrated the tumor bed. TCEs, conversely, act as pharmacological bridges, bypassing *ex vivo* manipulation to redirect circulating T cells against cancer cells *in situ*.

While the successes of these therapies have been transformative—creating long-term survivors among previously terminal patients with B-cell malignancies and melanoma—their broader application is severely restricted. The challenge now confronting the field is the stark dichotomy between outcomes in “liquid” hematologic cancers versus solid tumors. Solid tumors present a formidable fortress comprised of dense stroma, aberrant vasculature, and a metabolically hostile microenvironment that actively excludes and suppresses therapeutic T cells. This review will provide a rigorous examination of the molecular mechanisms, clinical nuances, and shared barriers facing these three modalities, arguing that the future of the field lies not in viewing them as competitors, but as complementary tools in a rationally designed combinatorial arsenal.

## CAR-T therapy: the synthetic biology revolution

Chimeric Antigen Receptor T-cell therapy is arguably the most clinically successful application of synthetic biology in human history. The assertion of unprecedented clinical efficacy is firmly grounded in long-term quantitative data from landmark trials. For instance, in the pivotal Phase 2 ZUMA-1 trial (NCT02348216) evaluating axicabtagene ciloleucel (CD19 CAR-T) in refractory large B-cell lymphoma, the objective response rate (ORR) was 83%, with a complete response (CR) rate of 58% (10, 11). This translates into genuine plateauing survival curves, where the 5-year overall survival (OS) rate reached 42.6%. Similarly, in the context of relapsed/refractory multiple myeloma, the CARTITUDE-1 trial (NCT03548207) evaluating ciltacabtagene autoleucel (BCMA CAR-T) demonstrated a remarkable ORR of 98% and a stringent CR of 83%, yielding a median progression-free survival (PFS) of 34.9 months (12).

### Molecular architecture and signaling kinetics

Physiological T cell activation requires a highly specific interaction between the T Cell Receptor (TCR) and a peptide-MHC complex. Tumors frequently downregulate MHC class I expression or components of the antigen processing machinery to become invisible to endogenous CD8+ T cells. The CAR construct bypasses this requirement by utilizing an antibody-derived single-chain variable fragment (scFv) to bind directly to unmodified cell surface proteins. The design of the intracellular signaling domain is critical to CAR function (13–15). First-generation CARs, containing only the CD3ζ signaling domain, supplied “Signal 1” (activation) upon antigen binding but failed to induce sufficient IL-2 production or prevent activation-induced cell death, leading to poor *in vivo* persistence. The breakthrough came with second-generation CARs, which incorporated a costimulatory endodomain—typically CD28 or 4-1BB (CD137)—in series with CD3ζ. This fusion provides simultaneous activation and costimulation (“Signal 2”). The choice of costimulatory domain profoundly influences T cell metabolic programming and effector function kinetics. CD28-based CARs (e.g., axicabtagene ciloleucel) characteristically exhibit rapid, intense expansion and potent immediate cytotoxicity, utilizing glycolytic metabolism like effector memory T cells. However, this intense activation can predispose them to faster exhaustion and shorter persistence. Conversely, 4-1BB-based CARs (e.g., tisagenlecleucel) promote mitochondrial biogenesis and oxidative phosphorylation, favoring the generation of central memory-like T cells with slower expansion kinetics but enhanced long-term persistence and resistance to exhaustion. These differences dictate clinical choices; for rapidly proliferating aggressive lymphomas, CD28 might be preferred, whereas for indolent diseases requiring long-term surveillance, 4-1BB may offer advantages (16–18). Building upon this dual-signaling model, third-generation CARs incorporate multiple costimulatory endodomains in tandem to integrate complementary functional attributes (19). However, the field has rapidly pivoted toward more complex structural modifications, resulting in fourth-generation CARs, commonly known as TRUCKs (T cells Redirected for Universal Cytokine Killing) or armored CARs. These specialized constructs are engineered with an inducible payload, utilizing antigen binding to trigger the localized release of potent cytokines, effectively remodeling the immunosuppressive tumor microenvironment (20, 21). Furthermore, a novel fifth generation of “cytokine-dependent” CARs has emerged, which integrates motifs from truncated cytokine receptors directly into the CAR backbone (22–24). Upon activation, these domains stimulate complementary natural survival pathways, such as the JAK-STAT signaling cascade, to prevent metabolic failure and terminal exhaustion.

While extrinsic barriers within the immunosuppressive TME present a robust challenge to CAR-T therapy, the intrinsic transcriptional, epigenetic, and metabolic landscape of the starting T-cell product establishes a fundamental, predetermined threshold for therapeutic efficacy and persistence (25, 26). A critical driver of failure lies in the starting product’s differentiation composition; infusing products enriched for highly differentiated effector T cells or effector memory T cells subsets—characterized by intense, glycolytic metabolism and rapid, but fleeting, cytotoxicity—often leads to rapid *in vivo* contraction and minimal persistence, particularly under chronic antigen stimulation (27). Conversely, enriching for stem cell memory (Tscm) and central memory (Tcm) subsets, which are metabolically pre-programmed for oxidative phosphorylation and robust self-renewal, has been shown to correlate directly with extended clinical responses. Furthermore, intrinsic design flaws can precipitate failure through cell-autonomous pathways, such as ligand-independent ‘tonic signaling’ (28, 29). This persistent activation, driven by high CAR surface expression levels or self-reactive spacer domains, can cause prolonged NFAT nuclear localization and subsequent epigenetic reprogramming into a fixed exhausted state, mediated by sustained expression of the TOX and NR4A transcription factor families, even in the absence of external checkpoint inhibition or TME metabolic stress (28, 30).

### Clinical triumphs and toxicity mechanisms

The clinical translation of chimeric antigen receptor T cell therapy has catalyzed a paradigm shift in the management of relapsed and refractory hematologic malignancies, transitioning diseases that were previously considered uniformly fatal into conditions with potential for durable, curative responses (16). The vanguard of this revolution has been CD19-directed autologous CAR-T cells. Long-term follow-up data from pivotal trials of axicabtagene ciloleucel and tisagenlecleucel in large B-cell lymphoma and pediatric acute lymphoblastic leukemia have demonstrated plateauing survival curves, with a significant fraction of patients maintaining minimal residual disease negativity for over a decade (16, 31, 32). Intriguingly, sophisticated clonal tracking and single-cell multi-omic analyses of these long-term survivors have revealed a biphasic persistence dynamic, where an initial massive expansion of CD8 positive cytotoxic effector cells is eventually replaced by a highly persistent, stable population of CD4 positive CAR-T cells exhibiting distinct central memory and helper-like transcriptional signatures (33, 34). Building upon the CD19 experience, the targeting of B-cell maturation antigen, or BCMA, has profoundly altered the therapeutic landscape for multiple myeloma (35). Products such as ciltacabtagene autoleucel and idecabtagene vicleucel have achieved unprecedented overall response rates and stringent complete response rates in heavily pretreated, penta-refractory myeloma populations (36, 37). However, the myeloma paradigm also highlights the persistent threat of relapse, driven either by the biallelic deletion and mutational downregulation of the BCMA locus or by the phenotypic exhaustion of the infused T cell product. To preempt these escape mechanisms, the field is rapidly advancing dual-targeting strategies and exploring novel antigens such as GPRC5D and FcRH5 to consolidate early remissions (38, 39).

Yet, the extraordinary cytolytic potency of CAR-T cells is inextricably linked to unique, potentially life-threatening toxicities that require highly specialized interdisciplinary management (22, 40). The most common and pathognomonic adverse event is cytokine release syndrome, a supraphysiologic systemic inflammatory response triggered immediately following target cell recognition and exponential CAR-T cell proliferation (41, 42). Modern mechanistic investigations have fundamentally redefined our understanding of cytokine release syndrome, shifting the focus from the CAR-T cells themselves to an intense crosstalk with the host innate immune system. Upon target engagement, CAR-T cells secrete copious amounts of interferon-gamma and tumor necrosis factor-alpha, which subsequently activate host bystander myeloid cells, particularly monocytes and macrophages. This activation frequently culminates in gasdermin-E mediated macrophage pyroptosis, a highly inflammatory form of programmed cell death that releases massive quantities of interleukin-1 beta and interleukin-6 into the systemic circulation. This interleukin-1 to interleukin-6 amplification axis drives the cardinal clinical manifestations of cytokine release syndrome, including high-grade fevers, vasodilatory hypotension, and capillary leak syndrome secondary to profound endothelial fenestration and angiopoietin-2 release (43–45). Consequently, the clinical management algorithm has evolved from purely reactive corticosteroid administration to preemptive interventions utilizing the interleukin-6 receptor antagonist tocilizumab and the interleukin-1 receptor antagonist anakinra, which effectively abrogate the cytokine storm without compromising the fundamental antileukemic efficacy of the CAR-T product (44, 45).

Distinct from but occasionally overlapping with cytokine release syndrome is immune effector cell-associated neurotoxicity syndrome, a complex encephalopathy that manifests as expressive aphasia, delirium, seizures, and in severe cases, fatal cerebral edema (43). The pathophysiology of immune effector cell-associated neurotoxicity syndrome is intensely scrutinized and appears to be driven by a confluence of systemic inflammation and localized blood-brain barrier disruption. High circulating levels of systemic cytokines, notably interleukin-15, interleukin-10, and granulocyte-macrophage colony-stimulating factor, facilitate the hyperpermeability of the cerebral endothelium, allowing both cytokines and activated mononuclear cells to traffic into the cerebrospinal fluid and central nervous system parenchyma (46, 47). Furthermore, cutting-edge single-cell transcriptomic profiling of the human brain has revealed low-level endogenous expression of CD19 on brain mural cells and pericytes. This suggests that a degree of on-target, off-tumor recognition by circulating CD19 CAR-T cells may directly trigger pericyte death and the subsequent structural collapse of the blood-brain barrier, providing a compelling mechanistic explanation for the disproportionately high rates of neurotoxicity observed with CD19-directed products compared to BCMA-directed therapies.

**Figure 1:**
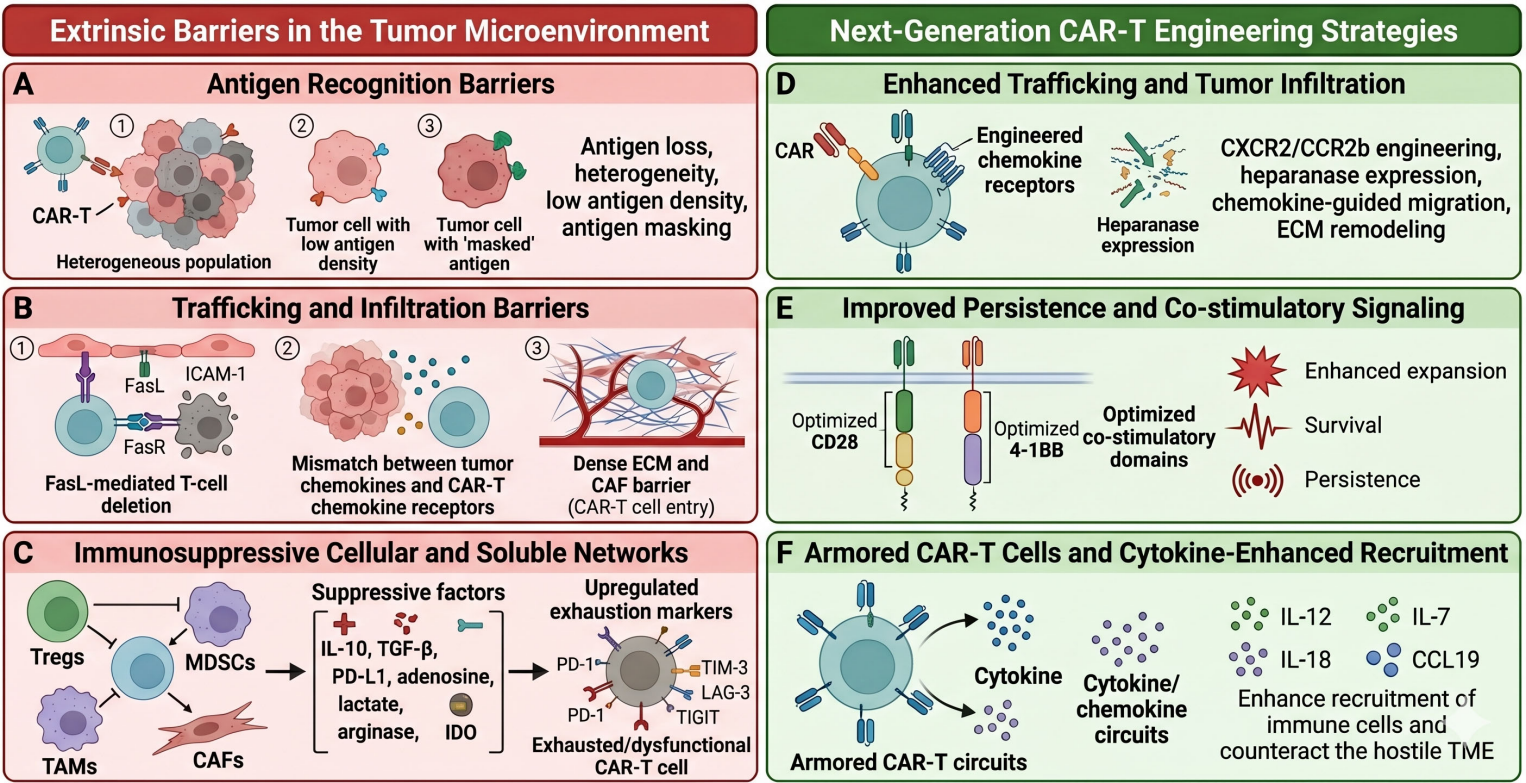
Extrinsic barriers in the solid tumor microenvironment and next-generation CAR-T cell engineering strategies. A schematic representation detailing the primary obstacles CAR-T cells encounter within the solid tumor microenvironment (left panels, red) and the corresponding rational engineering solutions designed to overcome them (right panels, green). **(A)** Antigen Recognition Barriers: Illustrates mechanisms of tumor antigen escape that impair CAR-T cell efficacy, including heterogeneous antigen expression, target cells with low antigen density, and spatial antigen masking. **(B)** Trafficking and Infiltration Barriers: Highlights the multifaceted physical and biochemical hurdles restricting T-cell tumor entry. These include (1) FasL-mediated CAR-T cell apoptosis induced by the tumor endothelium, (2) a mismatch between tumor-derived chemokines and CAR-T chemokine receptors preventing effective homing, and (3) the physical exclusion exerted by the dense ECM and CAFs. **(C)** Immunosuppressive Cellular and Soluble Networks: Depicts the hostile TME network where Tregs, MDSCs, TAMs, and CAFs release a milieu of suppressive factors. This microenvironment drives CAR-T cell exhaustion and dysfunction, characterized by the upregulation of inhibitory markers such as PD-1, TIM-3, LAG-3, and TIGIT. **(D)** Enhanced Trafficking and Tumor Infiltration: Demonstrates synthetic biology approaches to improve tumor penetration, including the transgenic expression of matched chemokine receptors and ECM-degrading enzymes like heparanase. **(E)** Improved Persistence and Co-stimulatory Signaling: Shows the optimization of intracellular co-stimulatory domains (e.g., CD28, 4-1BB) to deliver superior survival signals, thereby enhancing *in vivo* expansion, metabolic fitness, and long-term persistence. **(F)** Armored CAR-T Cells and Cytokine-Enhanced Recruitment: Illustrates the deployment of “armored” CAR-T circuits engineered to secrete transgenic cytokine/chemokine modules. These synthetic circuits actively counteract the immunosuppressive TME and recruit endogenous immune cells to orchestrate a broader anti-tumor response. CAF, cancer-associated fibroblast; CAR, chimeric antigen receptor; ECM, extracellular matrix; FasL, Fas ligand; FasR, Fas receptor; IDO, indoleamine 2,3-dioxygenase; IL, interleukin; LAG-3, lymphocyte-activation gene 3; MDSC, myeloid-derived suppressor cell; PD-1, programmed cell death protein 1; PD-L1, programmed death-ligand 1; TAM, tumor-associated macrophage; TGF-$\beta$, transforming growth factor-beta; TIGIT, T cell immunoreceptor with Ig and ITIM domains; TIM-3, T-cell immunoglobulin and mucin-domain containing-3; TME, tumor microenvironment; Treg, regulatory T cell.

Beyond acute inflammatory toxicities, the field is increasingly contending with delayed and chronic adverse events, most notably prolonged hematological toxicity (48–50). A substantial proportion of patients experience profound, prolonged cytopenias persisting for months after infusion, independent of the conditioning chemotherapy. This is currently understood to be a consequence of the inflammatory bone marrow microenvironment, where chronic interferon signaling suppresses hematopoietic stem and progenitor cell function, sometimes exacerbated by preexisting clonal hematopoiesis of indeterminate potential (51). To mitigate both acute and chronic toxicities, the next generation of synthetic biology is focusing on sophisticated control mechanisms. These include the implementation of pharmacologic ON and OFF switches, such as the use of the tyrosine kinase inhibitor dasatinib to temporarily halt CAR signaling complex assembly during periods of hyperinflammation, and the engineering of logic-gated genetic circuits that restrict profound activation strictly to the tumor microenvironment (**Figure 1**).

### The solid tumor impasse

The fundamental contrast between hematologic malignancies and solid tumors regarding antigen expression constitutes the primary barrier to translating the success of chimeric antigen receptor T cells into broader oncologic applications (15, 51). Hematologic malignancies possess lineage-restricted antigens, such as CD19, where the targeted ablation of the entire cellular lineage is clinically tolerable and manageable via supportive care like immunoglobulin replacement. Conversely, solid tumors predominantly express tumor-associated antigens rather than truly unique tumor-specific antigens. These targetable surface proteins are frequently shared with vital normal epithelia. Consequently, targeting antigens such as epidermal growth factor receptor, human epidermal growth factor receptor 2, or carcinoembryonic antigen with highly potent, synthetically activated T cells has historically precipitated catastrophic on-target off-tumor toxicities, manifesting clinically as fatal pulmonary or cardiopulmonary failure in early human trials (52–54). Furthermore, the inherent genomic instability and spatial evolution of solid tumors foster profound antigen heterogeneity. Even if a safe therapeutic target is identified, homogeneous and uniform expression across the entire primary tumor bulk and its distant metastases is exceedingly rare. This heterogeneous antigen landscape guarantees that the administration of a potent, monoclonal chimeric antigen receptor product will exert immense selective immunologic pressure on the tumor (55). This inevitably leads to the rapid eradication of antigen-positive clones and the unchecked outgrowth of antigen-negative clonal variants. This phenomenon of antigen escape is a dominant driver of clinical relapse and explicitly necessitates the development of sophisticated logic-gated synthetic circuits within the engineered T cells. Innovations such as synNotch receptors, which require the sequential recognition of multiple distinct antigens to initiate the transcription of the cytolytic chimeric antigen receptor, are currently being investigated to enhance tumor specificity, widen the therapeutic index, and critically mitigate the risk of singular antigen downregulation (55).

Beyond the complexities of target recognition, adoptively transferred T cells face an arduous and actively regulated journey from the systemic circulation into the solid tumor parenchyma. Solid tumors construct a formidable physical and chemokine barrier that actively excludes effector lymphocytes, creating what is clinically recognized as an immune-excluded tumor phenotype. The aberrant tumor vasculature is functionally tortuous, leaky, and frequently downregulates critical endothelial adhesion molecules, such as intercellular adhesion molecule 1 and vascular cell adhesion molecule 1, which are absolute biophysical prerequisites for T cell rolling, arrest, and extravasation (55, 56). Moreover, the tumor endothelium often undergoes specific phenotypic alterations, upregulating death ligands like Fas ligand, thereby creating a functional endothelial death cliff (57). This barrier selectively triggers apoptosis in infiltrating CD8 positive effector T cells while simultaneously permitting the entry of immunosuppressive regulatory T cells. Compounding this restrictive endothelial barrier is a profound localized chemokine mismatch. Instead of secreting T cell-attracting chemokines like CXCL9 and CXCL10, the solid tumor microenvironment characteristically secretes high levels of CXCL1, CXCL2, and CCL2 (57, 58). These chemokines establish a gradient that preferentially recruits immunosuppressive myeloid-derived suppressor cells and tumor-associated macrophages from the bone marrow. Even upon successful vascular extravasation, chimeric antigen receptor T cells immediately encounter a dense desmoplastic stroma orchestrated by cancer-associated fibroblasts (59). This stroma is characterized by a dense, highly cross-linked extracellular matrix rich in collagen and hyaluronic acid, establishing an area of remarkably high interstitial fluid pressure that physically traps T cells at the invasive margin and prevents physical conjugation with the malignant target cells deep within the tumor bed.

If a chimeric antigen receptor T cell successfully navigates the fibrotic stroma and contacts a malignant cell, it must execute its sustained cytolytic function within one of the most hostile metabolic and immunosuppressive environments in human pathophysiology. The solid tumor microenvironment is essentially a metabolic desert. The relentless Warburg metabolism of rapidly proliferating cancer cells depletes the interstitial fluid of essential nutrients, most notably glucose and critical amino acids such as glutamine, arginine, and tryptophan, the latter being actively catabolized by tumor-derived indoleamine 2,3-dioxygenase (58). Concurrently, the accumulation of metabolic waste products, including high concentrations of extracellular lactate and potassium, exerts a profound paralyzing effect on early T cell receptor signaling cascades and intracellular calcium flux. Operating under severe local hypoxia, chimeric antigen receptor T cells are forced into suboptimal metabolic states that fail to sustain the immense bioenergetic demands of continuous serial target killing and proliferation. This severe metabolic starvation is synergistically compounded by a dense resident network of cellular suppressors, including transforming growth factor-beta secreting regulatory T cells and polarized M2 macrophages, which continuously barrage the engineered T cells with inhibitory paracrine signals. Chronic, high-avidity antigen exposure in this localized immunosuppressive milieu inevitably drives a rapid transcriptional and epigenetic trajectory toward terminal T cell exhaustion. Driven by master regulatory transcription factors such as TOX and NR4A, this exhausted state is characterized by the sustained co-expression of multiple inhibitory checkpoint receptors, including programmed cell death protein 1, T cell immunoglobulin and mucin domain-containing protein 3, and lymphocyte-activation gene 3 (40, 60). Ultimately, this results in a fixed epigenetic landscape of profound functional anergy that is highly resistant to reversal by standard checkpoint blockade administration. Overcoming this multifaceted physical, metabolic, and cellular impasse currently forms the absolute frontier of synthetic immunology, driving the aggressive development of armored chimeric antigen receptors engineered to secrete matrix-degrading enzymes, express dominant-negative receptors designed to convert suppressive signals like transforming growth factor-beta into activating intracellular cascades, and utilize sophisticated CRISPR-Cas9 multiplex genome editing to permanently delete intrinsic exhaustion programming prior to patient infusion (60).

## TIL therapy: harnessing the natural polyclonal repertoire

The paradigm of harnessing the natural polyclonal repertoire has been clinically validated by recent regulatory milestones. The FDA approval of lifileucel for advanced, post-PD-1 melanoma was underpinned by the Phase 2 C-144–01 trial (NCT02360579) (61, 62). In this heavily pretreated cohort, a single infusion of lifileucel achieved an ORR of 31.4%, with a median OS of 13.9 months and a median duration of response that had not been reached at a median follow-up of 36.5 months, underscoring the durable nature of polyclonal T cell engraftment.

### Mechanisms of polyclonality and neoantigen targeting

The conceptual foundation of tumor-infiltrating lymphocyte therapy rests upon the exploitation of the naturally selected, highly diverse T cell receptor repertoire that has evolved directly within the patient in response to somatic mutagenesis. Unlike synthetic chimeric antigen receptors that monoclinally target a single, predefined surface construct, TILs leverage the physiological MHC antigen presentation machinery to survey the vast intracellular proteome. As tumors accrue non-synonymous passenger and driver mutations, they generate unique immunogenic peptides, termed neoantigens (63, 64). Because these neoantigens are strictly tumor-specific and bypass central tolerance mechanisms in the thymus, high-avidity T cells capable of recognizing them can exist and accumulate within the tumor bed. The profound clinical and biological advantage of this modality lies in its inherent polyclonality. A single therapeutic product expanded from a tumor fragment frequently comprises dozens to hundreds of distinct T cell clonotypes, capable of simultaneously recognizing a multitude of unique neoantigens. This highly multiplexed antigen recognition strategy essentially neutralizes the tumor’s ability to evade immune destruction through the downregulation or mutation of a single target antigen, a dominant escape mechanism that plagues monoclonal therapies. Furthermore, this broad repertoire can initiate a cascade of epitope spreading, where the destruction of tumor cells by the infused lymphocytes leads to the cross-presentation of new, previously unrecognized tumor antigens to the endogenous immune system, thereby providing a robust, self-amplifying immunological countermeasure to the spatial and temporal genomic heterogeneity that characterizes advanced solid malignancies (64).

### The biomanufacturing odyssey and phenotypic preservation

**Figure 2:**
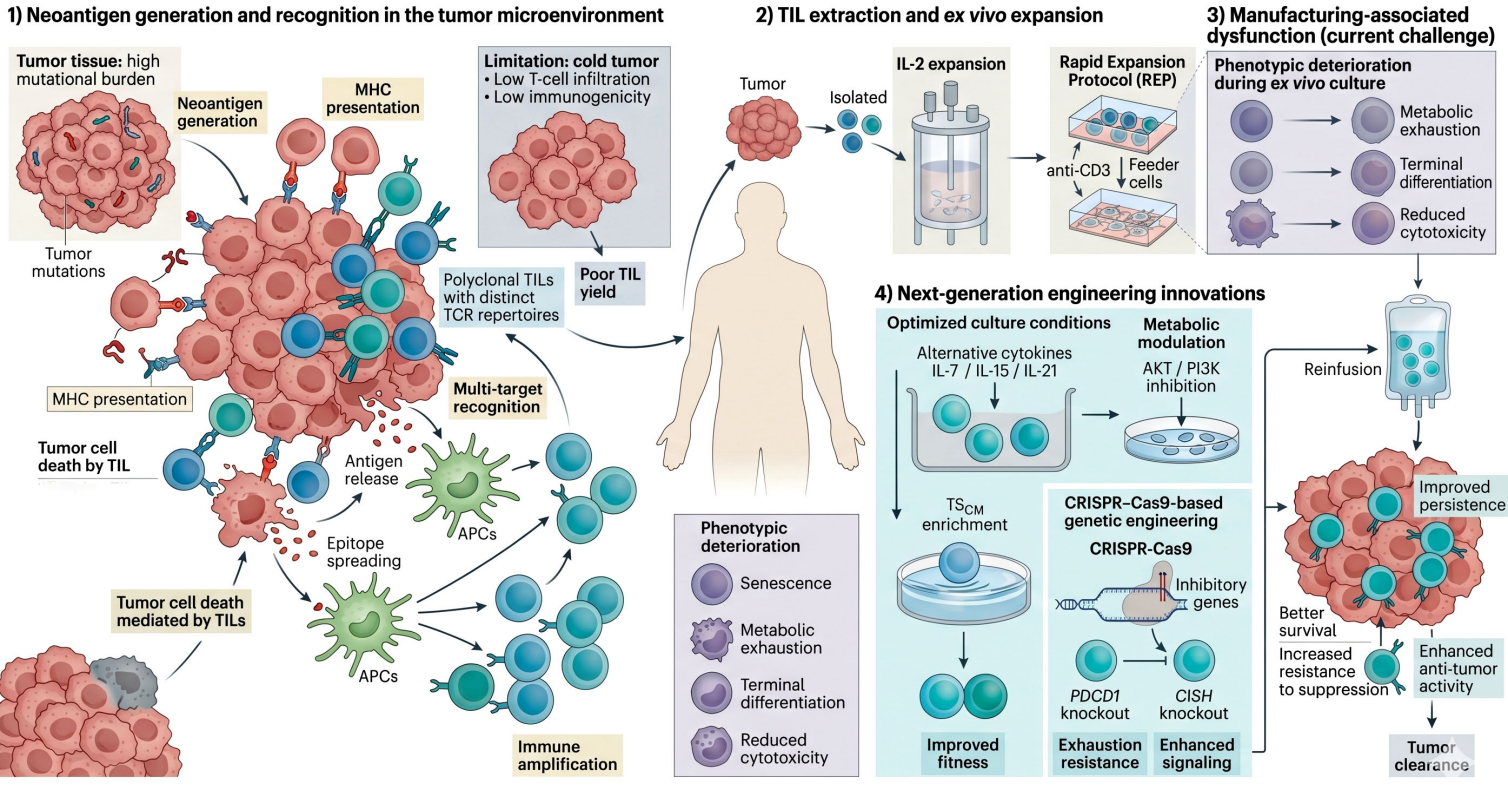
Detailed schematics of the TIL therapy workflow, current challenges, and engineering innovations. The illustration comprehensively details the TIL therapy pipeline, detailing the sequential steps from initial antigen discovery and recognition within the tumor microenvironment to *ex vivo* biomanufacturing, genetic engineering, and final reinfusion into the patient, while emphasizing critical mechanistic limitations and next-generation improvements. The process begins with neoantigen generation and presentation, where tumor mutations in the genomic landscape create unique intracellular proteins processed and presented as neoantigens via MHC molecules on the cell surface, followed by polyclonal TIL recognition where a diverse repertoire of T cells with distinct TCRs engages with multiple distinct neoantigens on tumor cells, enabling multi-target engagement. This multi-target recognition triggers an epitope spreading cascade, during which tumor cell death releases additional antigens taken up by antigen-presenting cells (APCs), leading to immune amplification and activation of new waves of T cells. In contrast, limitation in “cold tumors” highlights the significant challenge posed by non-inflamed tumors with low antigen load, low immunogenicity, and low T cell infiltration, which hinders effective TIL isolation. TILs are then extracted and progress through *ex vivo* bioprocessing, involving an initial IL-2-driven expansion and a subsequent Rapid Expansion Protocol (REP) with anti-CD3 stimulation and feeder cells to achieve massive proliferation. However, standard prolonged expansion in a REP can result in phenotypic deterioration, a critical limitation where T cells undergo negative outcomes including senescence, metabolic exhaustion, and terminal differentiation, ultimately reducing their cytotoxic capability. To address this, next-generation culture optimization strategies are employed, utilizing alternative cytokines (IL-7, IL-15, IL-21) and metabolic inhibitors (AKT/PI3K inhibition) to promote the formation and enrichment of stem cell memory T cells (T_SCM_). Simultaneously, genetic engineering leveraging precise CRISPR-Cas9 editing is used to knock out inhibitory genes such as $PDCD1$ or $CISH$ to enhance signaling, metabolic fitness, and resistance to exhaustion. The final product, consisting of persistent and engineered TILs, is then reinfused into the patient, where they overcome the suppressive microenvironment to effectively eliminate tumor cells and achieve enhanced persistence.

Despite its compelling biological rationale, the translation of this autologous repertoire into a viable clinical therapeutic is constrained by an immensely complex, artisan-level biomanufacturing odyssey (65). The classical derivation process requires the surgical excision of a macroscopic tumor lesion, followed by enzymatic or mechanical dissociation into micro-fragments. These fragments are subsequently cultured in supraphysiological concentrations of interleukin-2. This initial pre-rapid expansion protocol phase promotes the egress and proliferation of resident lymphocytes while simultaneously starving the malignant epithelium. However, the subsequent rapid expansion protocol phase, which utilizes anti-CD3 agonizing antibodies and irradiated allogeneic feeder mononuclear cells to drive exponential cellular proliferation, fundamentally alters the phenotypic and epigenetic landscape of the T cell product (66). Generating the tens of billions of cells required for a therapeutic dose inevitably exerts profound replicative stress, frequently driving the lymphocytes toward a terminally differentiated, senescent state characterized by critically shortened telomeres, profound metabolic exhaustion, and deep epigenetic scarring. Consequently, the vanguard of bioprocessing is currently shifting away from mere numerical expansion toward the preservation of a stem-like memory T cell phenotype. Researchers are actively modulating the *ex vivo* cytokine milieu, substituting continuous high-dose interleukin-2 exposure with rationally designed combinations of interleukin-7, interleukin-15, and interleukin-21, alongside the pharmacological inhibition of terminal differentiation pathways utilizing AKT or PI3K inhibitors (67). Furthermore, optimizing the manufacturing timeline to generate young tumor-infiltrating lymphocytes in a substantially compressed, closed-system automated workflow is critical. This not only yields a fitter, more cytolytic cellular product but also crucially minimizes the precarious time-to-vein interval for patients with rapidly progressing disease (**Figure 2**).

### Clinical milestones and next-generation genetic fortification

The clinical validation of tumor-infiltrating lymphocyte therapy has recently reached a historic inflection point, highlighted by regulatory approvals for advanced, immune checkpoint inhibitor-refractory metastatic melanoma (68). This milestone demonstrates remarkable, durable complete response rates in a patient population historically devoid of curative options, proving that solid tumors can indeed be eradicated by adoptive cell therapy. Beyond melanoma, robust efficacy is being demonstrated in other highly mutated or virally driven solid tumors, including mucosal human papillomavirus-positive cervical carcinomas and select cohorts of advanced non-small cell lung cancer (69).

Despite these successes in immunogenic malignancies, the application of standard, unmanipulated TIL therapy to immunologically ‘cold’ tumors—characterized by low tumor mutational burdens and sparse baseline immune infiltration—is significantly constrained (70, 71). Low-TMB malignancies fundamentally lack the diverse neoantigenic landscape necessary to provide a viably sufficiently reactive starting population of tumor-resident lymphocytes, making the initial *ex vivo* pre-rapid expansion protocol (pre-REP) viable for these patients (71, 72). Nevertheless, the broad application of unmanipulated tumor-infiltrating lymphocytes remains fundamentally limited by the intrinsic immunogenicity of the host tumor. Immunologically cold malignancies, characterized by low tumor mutational burdens and a paucity of pre-existing lymphocytic infiltration, simply do not yield a viable or sufficiently reactive starting population for *ex vivo* expansion (73). To overcome this limitation and fortify these cells against the profoundly immunosuppressive solid tumor microenvironment upon reinfusion, the field is rapidly embracing sophisticated next-generation genetic engineering. Consequently, next-generation TIL strategies often utilize gene editing, such as the transient knockout of PDCD1 or CISH via CRISPR-Cas9 during *ex vivo* expansion, to enhance effector function within the expanded polyclonal repertoire upon reinfusion. While highly effective at amplifying the cytolytic capacity and metabolic resilience of infiltrated T cells, it is critical to clarify that these intrinsic transcriptional modifications do not inherently correct defects in T-cell trafficking or recruitment to immune-desert tumors. Rather, they serve to ‘arm’ the existing infiltrated population against the immunosuppressive and metabolic barriers of the established microenvironment (74, 75).

## T cell engagers: the molecular bridge

In the solid tumor landscape, TCEs have achieved critical quantitative breakthroughs. Tebentafusp, a gp100×CD3 bispecific, demonstrated a definitive survival benefit in metastatic uveal melanoma (NCT03070392), improving the 1-year OS from 59% to 73% (76–78). More recently, tarlatamab, a DLL3×CD3 TCE, achieved an ORR of 40% and a median OS of 14.3 months in heavily pretreated small-cell lung cancer (DeLLphi-301, NCT05060016), confirming the viability of the TCE modality beyond hematologic malignancies (79).

### Structural evolution and the induced immunological synapse

The structural evolution of TCEs represents a sophisticated engineering trajectory aimed at optimizing the delicate balance between anti-tumor potency, serum half-life, and systemic safety. The paradigm originated with the first-generation tandem single-chain variable fragment (scFv) format, exemplified by blinatumomab. These 55 kDa molecules, lacking an Fc domain, facilitate a rapid and potent cytolytic synapse but are characterized by an exceptionally short circulatory half-life, necessitating continuous intravenous infusion to maintain therapeutic concentrations above the renal filtration threshold (80).

To circumvent these pharmacokinetic limitations, the lineage progressed toward Fc-extended, IgG-like formats. These contemporary constructs utilize human IgG scaffolds or heterodimeric Fc regions to leverage neonatal Fc receptor (FcRn) recycling, extending the half-life to several days and permitting intermittent dosing. Crucially, to prevent non-specific systemic immune activation via Fcγ -bearing myeloid cells, these scaffolds incorporate precise Fc-silencing mutations, such as the L234A/L235A (LALA) or N297G substitutions, ensuring that T-cell activation remains strictly dependent on tumor-antigen-mediated crosslinking (81, 82).

The structural frontier has recently shifted toward conditionally active ‘masked’ TCEs (pro-TCEs) and affinity-tuned architectures. Masked formats utilize peptide covers linked by tumor-enriched protease-cleavable substrates, which sterically hinder the CD3-binding arm in systemic circulation but unleash full potency within the malignant interstitium, thereby widening the therapeutic index (83, 84). Simultaneously, engineering efforts have transitioned from high-affinity CD3 binding to affinity-tuned domains; by utilizing low-affinity CD3 arms alongside high-valency tumor-binding domains, these constructs favor preferential accumulation in tumors while mitigating the peak cytokine release syndrome (CRS) associated with initial systemic T-cell engagement (85).

Finally, the structural evolution culminates in trispecific and multispecific architectures. These advanced platforms integrate ‘Signal 1’ (CD3) with ‘Signal 2’ (costimulatory domains such as CD28 or 4-1BB) or dual tumor-associated antigens in a single polypeptide chain. By mimicking the integrated signaling dynamics of a second-generation CAR-T cell in trans, these multispecific engagers provide the necessary costimulatory support to sustain T-cell metabolic fitness and prevent the induction of anergy within the suppressive solid tumor microenvironment.

### Trispecifics and the costimulatory frontier in solid tumors

The most formidable barrier facing the T cell engager platform is achieving durable efficacy in the metabolically hostile and profoundly immunosuppressive microenvironment of solid tumors. Canonical bispecific engagers provide a potent activating signal via CD3 crosslinking, functionally equivalent to Signal 1 in classical immunology. However, they entirely lack the capacity to deliver a concurrent costimulatory signal, or Signal 2. In the context of a chronic, highly suppressive solid tumor microenvironment, the provision of isolated Signal 1 inevitably drives the recruited T cells into a state of deep anergy and terminal functional exhaustion (86). Furthermore, this lack of costimulation severely impairs the generation of long-lived memory T cell populations, rendering the patient highly susceptible to disease relapse once the pharmacological agent is cleared from the circulation.

**Figure 3:**
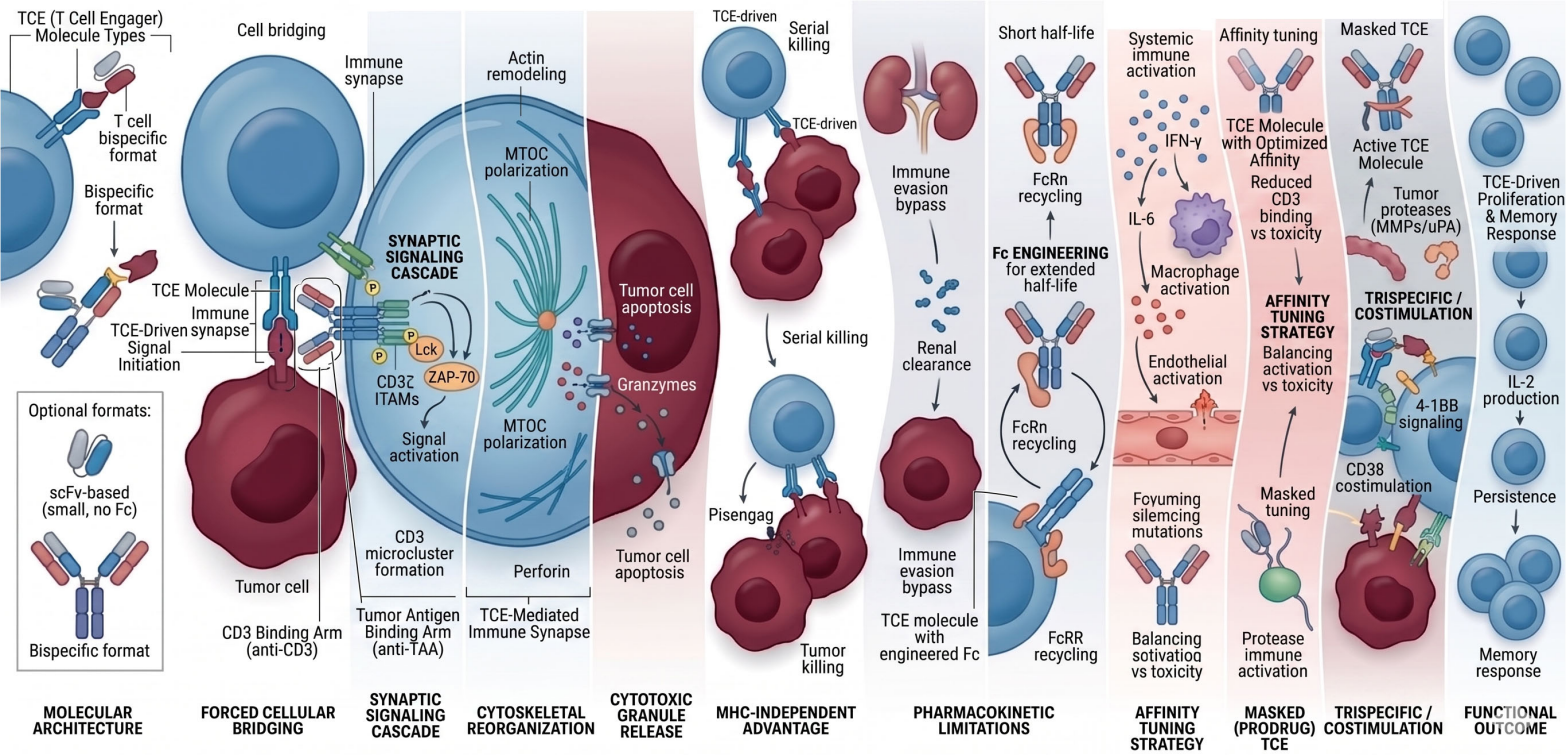
Integrated mechanisms of action, pharmacokinetic (PK) limitations, and engineering strategies for TCEs. T cell engagers (TCEs) are therapeutic molecules designed to bridge T cells and target cells (typically tumor cells) for potent cytotoxicity. The diagram illustrates common TCE formats under Molecular Architecture, including small molecules based on single-chain variable fragments (scFv, lacking an Fc domain) and larger, IgG-like molecules (with an Fc domain). These molecules initiate action through Forced Cellular Bridging, physically connecting CD3 on a T cell and a tumor antigen to form an Immune synapse, notably bypassing natural MHC-mediated antigen presentation for a direct MHC-Independent Advantage. Detailed vertical panels describe the T cell-centric cellular events: Synaptic Signaling Cascade details the intracellular activation, where binding leads to CD3 microcluster formation, phosphorylation of CD3ζ ITAMs, and activation of Lck and ZAP-70; Cytoskeletal Reorganization shows subsequent polarization of the microtubule-organizing center (MTOC) and actin towards the synapse; Cytotoxic Granule Release depicts the polarized release of perforin and granzymes to induce tumor cell apoptosis. Robust TCE activation enables a single T cell to perform Serial Killing of multiple tumor cells sequentially. The right side of the diagram addresses key limitations and optimization approaches: • PK Limitations: Highlights challenges such as short half-life, significant Renal clearance, and risks of capillary leak. Strategies to address these include Fc Engineering (Extended Half-Life) via the FcRn recycling pathway, which also often incorporates mutations for FcγR silencing to prevent off-target activation of cells like macrophages or endothelium (minimizing Fc silencing). • Affinity Tuning & Masking: Approaches to balance therapeutic activation and systemic toxicity like Cytokine Release Syndrome (CRS), characterized by an IL-6 surge, endothelial activation, and capillary leak, include Affinity Tuning Strategies and the use of Masked (Pro-drug) TCEs that employ a Peptide cover for protease activation within the protease-rich tumor microenvironment (e.g., by MMPs/uPA).• Trispecific/Costimulation: Demonstrates next-generation Trispecific (Pro-drug) TCEs that integrate costimulatory signaling (e.g., via CD28 or 4-1BB) with CD3 activation and tumor antigen binding for a core, integrated signal.Ultimately, successful TCE therapy leads to desired Functional Outcomes, including memory T cell responses, enhanced T cell proliferation, IL-2 production, and long-term persistence. Summary horizontal and vertical labels indicate major mechanistic and optimization domains across the continuous landscape.

To overcome this fundamental biological limitation, the absolute frontier of engager technology has evolved from bispecific to trispecific and multispecific architectures. These next-generation molecules are engineered to simultaneously bind a tumor-associated antigen, CD3, and a critical costimulatory receptor on the T cell surface, most notably CD28 or the tumor necrosis factor receptor superfamily member 4-1BB (87). By integrating these specific costimulatory domains, trispecific engagers meticulously mimic the highly successful intracellular signaling dynamics of second-generation chimeric antigen receptors, completely in trans. The incorporation of CD28 binding forcefully drives interleukin-2 production, enhances T cell proliferation, and shifts the metabolic profile to sustain cytotoxicity in nutrient-deprived environments (88). Alternatively, integrating a 4-1BB binding arm promotes mitochondrial biogenesis, dramatically enhances the resistance to exhaustion-induced apoptosis, and strongly favors the differentiation of central memory T cells. By transforming the transient, pharmacologically induced synapse into a fully competent, costimulated immunological event, these advanced multi-specific constructs represent a critical leap forward in empowering endogenous T cells to dismantle the solid tumor fortress, ensuring both immediate tumor eradication and durable, long-term immune surveillance (89) (**Figure 3**).

## The great barriers: TME and antigen escape

### The physical and metabolic fortress of the solid tumor stroma

The transition of T cell-driven immunotherapies from hematologic malignancies to solid tumors fundamentally requires conquering the tumor microenvironment, a highly evolved, pathophysiological ecosystem designed to actively exclude and neutralize immune effector cells. The first layer of this defense is a profound biophysical barrier known as desmoplasia. Orchestrated primarily by cancer-associated fibroblasts, this process involves the massive deposition of a dense, highly cross-linked extracellular matrix rich in fibrillar collagens, fibronectin, and hyaluronan. This dense stromal network significantly elevates interstitial fluid pressure, creating a severe biophysical impediment that physically traps infiltrating T cells at the tumor periphery, culminating in the clinically recognized immune-excluded phenotype (90). Concurrently, the tumor vasculature is inherently chaotic, structurally abnormal, and functionally leaky. These vessels frequently downregulate critical endothelial adhesion molecules such as intercellular adhesion molecule 1 and vascular cell adhesion molecule 1, while upregulating death ligands like Fas ligand, thereby selectively inducing apoptosis in extravasating cytotoxic T cells while permitting the entry of immunosuppressive populations (91).

Beyond physical exclusion, the solid tumor microenvironment represents a fundamentally hostile metabolic desert that actively paralyzes T cell effector functions. Driven by the *Warburg* effect, rapidly proliferating malignant cells uncouple glycolysis from oxidative phosphorylation, consuming vast quantities of local glucose. This extreme nutrient competition starves infiltrating T cells of the primary bioenergetic substrate required to fuel clonal expansion and the synthesis of effector cytokines like interferon-gamma (92). Furthermore, the tumor actively depletes the microenvironment of essential amino acids critical for T cell survival. The upregulation of arginase-1 and indoleamine 2, 3-dioxygenase by both tumor cells and resident myeloid cells rapidly catabolizes local arginine and tryptophan, respectively. The resulting accumulation of toxic metabolites like kynurenine directly binds to the aryl hydrocarbon receptor on T cells, actively suppressing their proliferation and promoting their differentiation into regulatory phenotypes (93).

**Figure 4:**
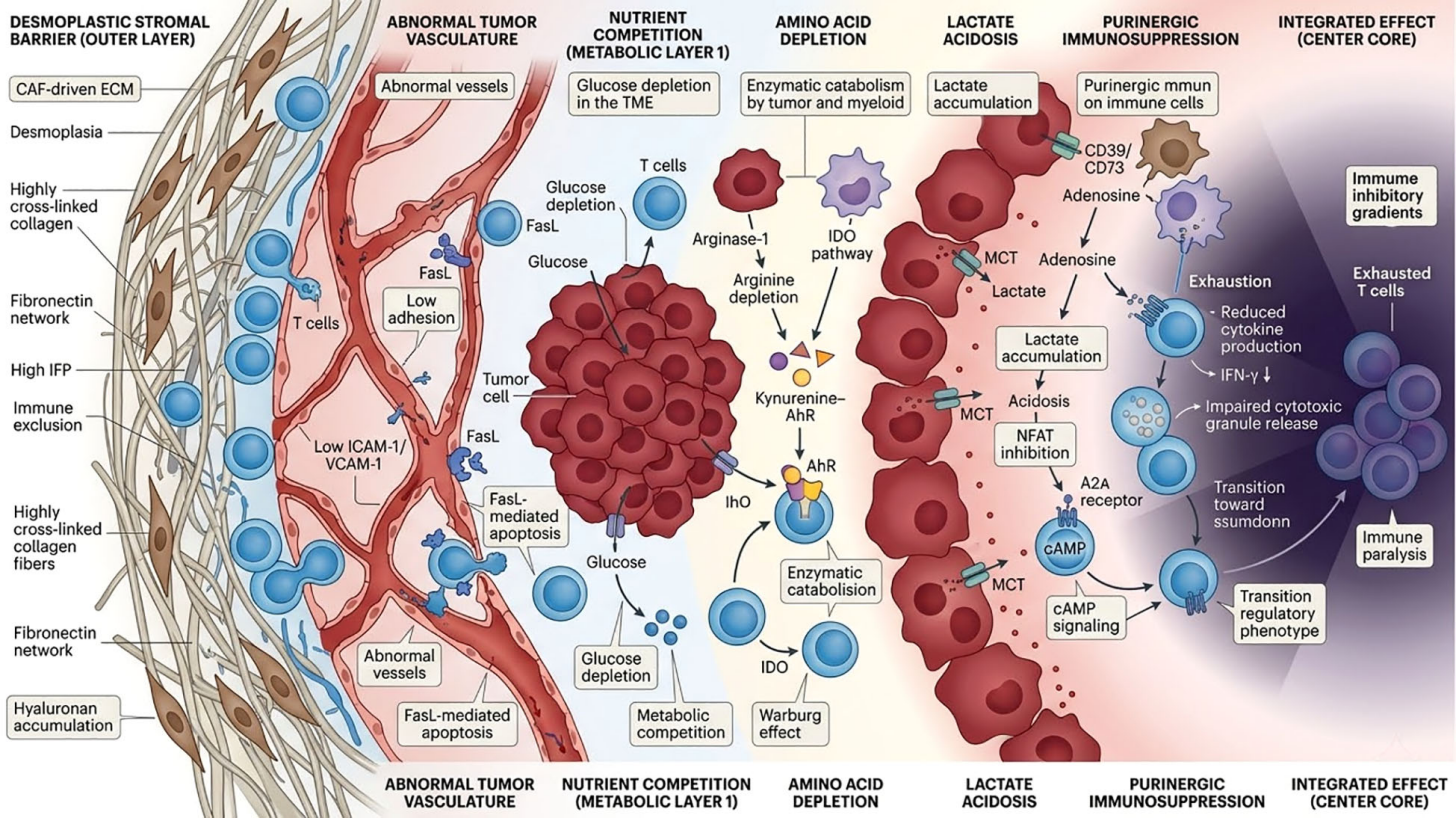
The multiscale barriers of the solid tumor microenvironment that collectively prevent effective anti-tumor immunity. The overall organization is concentric, moving from an outer stromal fortress of CAF-driven matrix, desmoplasia, and cross-linked collagen that causes high interstitial fluid pressure and immune cell exclusion, to an inner metabolic landscape of nutrient competition and signal suppression. Following infiltration, abnormal vasculature prevents T-cell exit and function through low adhesion due to low ICAM-1 and VCAM-1 expression, and the expression of death-inducing Fas ligand. The middle metabolic layers detail glucose competition from tumor cells and the Warburg effect, which leads to glucose depletion and deprives T-cells of energy, and multiple immunosuppressive metabolic pathways. Amino acid depletion involves Arginase-1 and IDO pathways degrading essential arginine and tryptophan and generating immunosuppressive kynurenine metabolites. Lactate acidosis is detailed with lactate accumulation and export via MCT leading to NFAT inhibition in T-cells. Purinergic immunosuppression involves the CD39/CD73 axis converting ATP to adenosine which binds to A2A receptors to increase cAMP signaling and drive exhaustion and a regulatory phenotype. The whole system culminates in the central core integrated effect where multiple immune inhibitory gradients overlap to produce advanced T-cell exhaustion and terminal differentiation characterized by reduced cytokine production and cytotoxic granule release and a functional collapse into immune paralysis.

Simultaneously, the massive glycolytic flux of the tumor generates copious amounts of lactic acid, which is actively exported into the extracellular space via monocarboxylate transporters. This profound localized acidosis directly impairs the enzymatic function of critical intracellular T cell signaling cascades, blunting the activation of the nuclear factor of activated T cells and severely diminishing cytolytic degranulation (94). Adding to this metabolic suppression is the purinergic signaling axis. The hypoxic tumor microenvironment drives the coordinate upregulation of the ectonucleotidases CD39 and CD73 on both tumor and regulatory cells, which sequentially hydrolyze extracellular ATP into immunosuppressive adenosine. This adenosine binds specifically to A2A receptors on the surface of infiltrating T cells, triggering an accumulation of intracellular cyclic AMP that potently and rapidly shuts down T cell receptor signaling and effector function, effectively inducing a state of deep metabolic anergy (95) (**Figure 4**).

### The immunosuppressive cellular network and T cell exhaustion

Even if engineered or naturally selected T cells successfully navigate the physical and metabolic barriers of the stroma, they must survive a dense, actively suppressive cellular network. Solid tumors secrete a highly specific gradient of chemokines, such as CCL2, CXCL1, and CXCL2, which preferentially recruit immature myeloid cells from the bone marrow. Upon entering the tumor microenvironment, these cells are polarized into myeloid-derived suppressor cells and M2-like tumor-associated macrophages (96). These highly suppressive myeloid populations blanket the tumor bed, continuously secreting potent inhibitory cytokines, most notably transforming growth factor-beta and interleukin-10. Transforming growth factor-beta is arguably the master regulator of immune exclusion and suppression in solid tumors; it not only drives the stromal fibrosis but also binds directly to receptors on CD8 positive T cells, triggering SMAD-mediated transcriptional programs that directly repress the expression of critical cytotoxic molecules like perforin and granzyme (97). Furthermore, the tumor actively recruits FoxP3 positive regulatory T cells, which act as dominant sinks for the homeostatic cytokine interleukin-2, thereby depriving effector T cells of crucial survival signals while simultaneously mediating contact-dependent suppression.

Immersed in this highly inhibitory paracrine milieu, and subjected to relentless, high-avidity stimulation by persistent tumor antigens, infiltrating T cells inevitably undergo a profound developmental trajectory toward terminal exhaustion. Exhaustion is not merely a transient state of cellular fatigue, but rather a distinct, epigenetically wired differentiation program driven by continuous T cell receptor engagement. At the molecular level, chronic antigen exposure triggers the sustained expression of master regulatory transcription factors, primarily TOX and members of the NR4A family (98). These transcription factors orchestrate a massive remodeling of the chromatin landscape, establishing a fixed epigenetic state characterized by the sustained, high-level co-expression of multiple inhibitory checkpoint receptors, including programmed cell death protein 1, T cell immunoglobulin and mucin domain-containing protein 3, lymphocyte-activation gene 3, and TIGIT (99–102).

**Figure 5:**
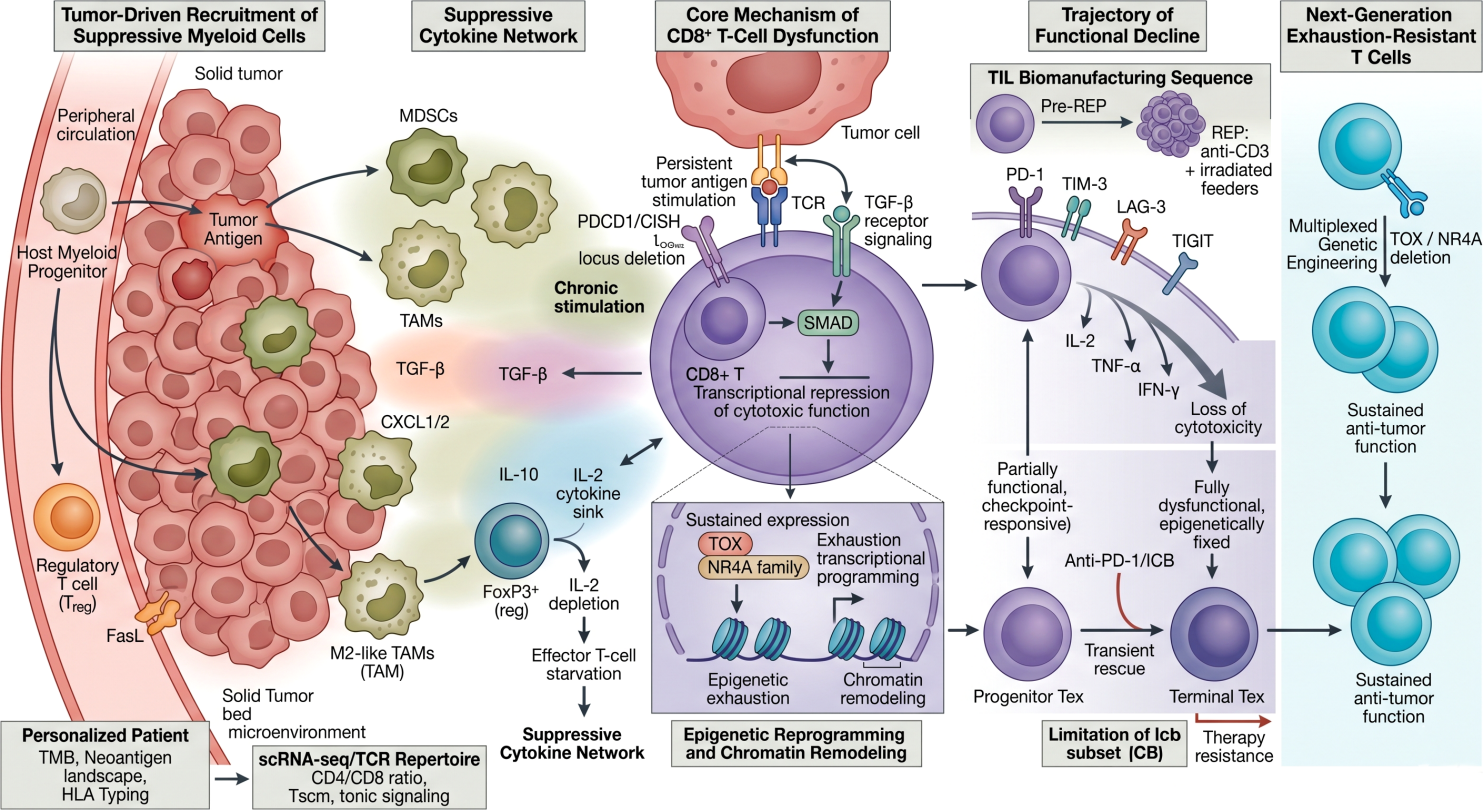
The tumor microenvironment creates an active immunosuppressive ecosystem that recruits myeloid populations and drives progressive T cell exhaustion through epigenetic reprogramming. The diagram illustrates the coordinated cellular and molecular networks within a solid tumor that facilitate immune evasion. Tumors establish an immunosuppressive milieu by actively orchestrating the recruitment of regulatory populations. Tumor cells secrete chemokines, such as CCL2 and CXCL1/2, which recruit immature myeloid cells from peripheral circulation into the tumor parenchyma. Once inside the tumor microenvironment (TME), these infiltrating myeloid cells differentiate into potent immunosuppressive populations, including Myeloid-Derived Suppressor Cells (MDSCs) and M2-like Tumor-Associated Macrophages (TAMs). These myeloid populations, along with FoxP3+ regulatory T (Treg) cells, secrete inhibitory cytokines like TGF-$\beta$ and IL-10, establishing a suppressive diffusion gradient. Treg cells further inhibit effector T cells by acting as an IL-2 cytokine sink, leading to local IL-2 depletion and starvation of effector cells. Centrally, the diagram focuses on the mechanisms driving CD8+ T cell dysfunction. TGF-$\beta$ signaling through its receptor activates the SMAD signaling cascade, resulting in the transcriptional repression of cytotoxic functions. However, a major driver is Chronic Stimulation resulting from persistent tumor antigen engagement with the T cell receptor (TCR). This continuous activation drives a unique transcriptional and Epigenetic exhaustion program. The sustained expression of critical transcription factors, including TOX and the NR4A family, initiates profound Chromatin remodeling, locking the T cell in a fixed, dysfunctional state. Exhausted T cells progressively upregulate Checkpoint receptors on their surface, including PD-1, TIM-3, LAG-3, and TIGIT, leading to a characteristic trajectory of Functional decline marked by the sequential loss of IL-2, TNF-$\alpha$, and finally IFN-$\gamma$ production, culminating in a loss of cytotoxicity. The illustration differentiates two major exhausted T cell populations: Progenitor Tex (partially functional) and Terminal Tex (fully dysfunctional). This heterogeneity underlies a critical Limitation of immune checkpoint blockade (ICB) therapies, such as anti-PD-1; ICB only offers transient rescue of Progenitor Tex cells, while the cells ultimately progress to the Terminal Tex state, representing a form of therapy resistance. To overcome these limitations, next-generation engineering strategies propose the development of Exhaustion-resistant T cells by genetically deleting key driving pathways like TOX/NR4A, enabling sustained anti-tumor function under chronic stimulation conditions.

As T cells progress from progenitor exhausted states to terminal exhaustion, they sequentially lose the capacity to secrete interleukin-2, tumor necrosis factor-alpha, and eventually interferon-gamma, culminating in a complete cessation of cytolytic activity (103). Crucially, cutting-edge epigenetic profiling has revealed that while immune checkpoint blockade can transiently reinvigorate progenitor exhausted T cells, the terminally exhausted state is locked in by profound DNA methylation and chromatin inaccessibility (104). This explains why adoptively transferred chimeric antigen receptor T cells or tumor-infiltrating lymphocytes often exhibit spectacular initial expansion and tumor regression, only to succumb to deep, irreversible exhaustion weeks later, underscoring the absolute necessity for next-generation genetic engineering strategies designed to permanently delete exhaustion-driving transcription factors prior to infusion (105) (**Figure 5**).

### The Darwinian dynamics of antigen escape and tumor plasticity

The ultimate and most insidious barrier to curative T cell immunotherapy is the profound genomic and phenotypic plasticity of the tumor itself, a phenomenon that drives antigen escape. The fundamental mechanism of antigen escape is rooted in Darwinian evolutionary principles. When a patient is treated with a highly potent, monoclinal targeted therapy, such as a chimeric antigen receptor T cell or a bispecific T cell engager directed against a single antigen like CD19 or BCMA, an immense selective immunologic pressure is applied to the tumor mass. While most antigen-positive malignant cells are rapidly eradicated, any pre-existing or *de novo* mutated tumor clone lacking the targeted antigen is granted an absolute survival advantage. These rare, antigen-negative variants rapidly expand to repopulate the tumor space, manifesting clinically as a devastating, target-negative relapse (106).

The molecular mechanisms facilitating this evasion are remarkably diverse and sophisticated. The most direct mechanism involves somatic mutations, such as frameshifts or non-sense mutations, leading to the biallelic loss of the gene encoding the target antigen. Alternatively, tumors may employ post-transcriptional evasion strategies, famously exemplified by the alternative splicing of the CD19 messenger RNA (107). By excising exon 2, which encodes the specific extracellular epitope recognized by the clinically approved CAR construct, the leukemia cell successfully maintains vital intracellular CD19 signaling while rendering itself completely invisible to the engineered T cells. Beyond structural alterations of the target, tumors frequently exploit profound lineage plasticity to evade destruction. Under the intense pressure of B-cell directed therapies, leukemic clones have been documented to undergo a complete epigenetic reprogramming, undergoing a lineage switch from a lymphoid to a myeloid phenotype, thereby shedding the entire suite of B-cell antigens simultaneously while retaining their malignant proliferative capacity (108).

For major histocompatibility complex-dependent therapies, such as tumor-infiltrating lymphocytes or endogenous T cells stimulated by checkpoint blockade, antigen escape frequently occurs via the systemic disruption of the antigen processing and presentation machinery. Solid tumors routinely acquire truncating mutations or deletions in the beta-2 microglobulin gene, which is an absolute requisite for the stable cell surface expression of all human leukocyte antigen class I molecules (109). Similarly, epigenetic silencing or mutational inactivation of the transporter associated with antigen processing genes completely abrogates the transport of immunogenic neo-peptides into the endoplasmic reticulum (110). By globally downregulating major histocompatibility complex class I expression, the tumor effectively blinds the entire endogenous and adoptively transferred T cell receptor repertoire, rendering even the most robust polyclonal immune response completely impotent. This profound capacity for evolutionary evasion definitively dictates that the future of curative cellular immunotherapy cannot rely on single target modalities but must inherently incorporate highly multiplexed targeting strategies and combinatorial approaches designed to corner the tumor evolutionarily and preempt the emergence of escape variants (111).

## Biomarker-driven patient stratification and personalized therapy selection

The clinical utility of CAR-T, TILs, and TCEs is increasingly contingent upon the precise stratification of patients based on the distinct immunological and genomic landscapes of their tumors. Transitioning from a modality-centric to a patient-tailored approach requires the integration of high-dimensional biomarkers to navigate the “hot,” “excluded,” and “cold” tumor immune phenotypes (**Table 1**).

**Table 1:** Rational selection of t cell modalities based on tumor immune phenotypes.

Tumor immune phenotype	Immunological characteristics	Key biomarkers for selection	Optimal t cell strategy	Proposed combinatorial intervention
"Hot" (Inflamed)	High TIL infiltration; robust IFN-γ signaling; high PD-L1 expression.	High TMB; TCF-1+ progenitor Tex (scRNA-seq); diverse TCR repertoire.	TIL Therapy; Standard CAR-T; TCEs.	ICB (Anti-PD-1) to sustain effector function.
Immune-Excluded	T cells present at the invasive margin but sequestered in the stroma.	High TGF-$\beta$; dense FAP+ fibroblast activity; stromal collagen deposition.	TCEs (to bridge stromal gaps); CAR-T with collagenase expression.	TGF-β inhibitors; FAP-targeted CARs or TCEs.
"Cold" (Desert)	Complete absence of T cells within the tumor parenchyma or margins.	Low TMB; lack of CXCL9/10/13 expression; defective DC priming.	Armored CAR-T (expressing IL-12/CCL19); TCEs + Priming agents.	Oncolytic viruses or TLR agonists to "heat up" the TME.

CAR-T, chimeric antigen receptor T cell; CCL19, C-C motif chemokine ligand 19; CXCL, C-X-C motif chemokine ligand; DC, dendritic cell; FAP, fibroblast activation protein; ICB, immune checkpoint blockade; IFN-γ, interferon gamma; IL-12, interleukin 12; PD-1, programmed cell death protein 1; PD-L1, programmed death-ligand 1; scRNA-seq, single-cell RNA sequencing; TCEs, T cell engagers; TCF-1, T cell factor 1; TCR, T cell receptor; Tex^prog, progenitor exhausted T cells; TGF-β, transforming growth factor beta; TIL, tumor-infiltrating lymphocyte; TLR, Toll-like receptor; TMB, tumor mutational burden; TME, tumor microenvironment.

A critical determinant for TIL therapy success is the pre-existing neoantigen landscape and TCR repertoire diversity. Advanced scRNA-seq has revealed that the presence of progenitor exhausted T cells, characterized by TCF-1 expression, is a superior predictor of durable response compared to absolute TIL quantification (112, 113). Furthermore, longitudinal TCR repertoire profiling allows clinicians to distinguish between bystander T cells and true tumor-reactive clones, ensuring that the starting material for TIL expansion possesses sufficient clonal breadth to counter immunoediting and antigen drift. For CAR-T and TCE interventions, the spatial architecture of TME and the presence of “immune-excluded” barriers are paramount. In tumors where T cells are sequestered in the stroma by dense collagen matrices or TGF-β-driven fibroblast activity, TCEs may offer a competitive advantage by physically bridging peripheral T cells to the tumor boundary (114). Conversely, in “immune-desert” or “cold” tumors, the lack of endogenous priming necessitates the use of “armored” CAR-T cells capable of initiating *de novo* immune recruitment through localized chemokine secretion or logic-gated synthetic circuits. Furthermore, genomic biomarkers, including tumor mutational burden and HLA heterozygosity, serve as foundational filters for modality selection. The loss of heterozygosity at the HLA locus is a frequent mechanism of resistance that specifically impairs TIL and TCR-T efficacy while potentially leaving CAR-T—which functions in an HLA-independent manner—as the only viable cellular option (115). By integrating these multi-omic layers, personalized immunotherapy can move beyond empirical selection toward a rationally stratified clinical framework.

## Future directions: engineering and combinatorial synergy

The clinical maturation of CAR-T, TILs, and TCEs has provided a robust, multi-modal foundation for the next generation of precision oncology. As synthesized in **Table 2**, the landmark clinical successes and quantitative milestones achieved by these three platforms—ranging from curative plateaus in hematologic malignancies to emerging survival benefits in recalcitrant solid tumor niches—validate the fundamental premise of direct T-cell engagement. However, the persistent challenges of physical exclusion, metabolic starvation, and antigenic heterogeneity in most solid tumors suggest that the maximal therapeutic ceiling of any single-agent approach may have been reached. Consequently, the frontier of immunotherapy is pivoting from platform-specific optimization toward a paradigm of ‘Engineering and Combinatorial Synergy.’ By strategically integrating these T-cell directed agents with external modulators designed to functionally remodel the hostile tumor microenvironment, we can begin to dismantle the defensive barriers of ‘cold’ tumors, a strategy exemplified by the rational deployment of oncolytic virotherapy to ignite the local immune landscape.

**Table 2:** Landmark clinical evidence across t cell engaging modalities: tumor type, intervention, and quantitative outcomes.

Tumor type	Modality	Agent (Target)	Trial phase & ClinicalTrials.gov ID	Key clinical outcomes (ORR, CR, PFS, OS)
Large B-cell Lymphoma (LBCL)	CAR-T	Axicabtagene ciloleucel (CD19)	Phase 2 (ZUMA-1) NCT02348216	ORR: 83%; CR: 58% 5-year OS: 42.6%
Multiple Myeloma (MM)	CAR-T	Ciltacabtagene autoleucel (BCMA)	Phase 1b/2 (CARTITUDE-1) NCT03548207	ORR: 98%; sCR: 83% Median PFS: 34.9 months Median OS: Not reached
Advanced Melanoma	TIL	Lifileucel (Polyclonal)	Phase 2 (C-144-01) NCT02360579	ORR: 31.4% Median OS: 13.9 months
Cervical Cancer	TIL	LN-145 (Polyclonal)	Phase 2 (C-145-04) NCT03108495	ORR: 44.4%; CR: 11.1%
Uveal Melanoma	TCE	Tebentafusp (gp100 × CD3)	Phase 3 NCT03070392	1-year OS: 73% (vs. 59% control Median OS: 21.7 months
Small-Cell Lung Cancer (SCLC)	TCE	Tarlatamab (DLL3 × CD3)	Phase 2 (DeLLphi-301) NCT05060016	ORR: 40% Median OS: 14.3 months Median DOR: 9.7 months
B-cell ALL	TCE	Blinatumomab (CD19 × CD3)	Phase 3 (TOWER) NCT02013167	CR/CRh: 43.6% (vs. 24.6% chemo) Median OS: 7.7 months (vs. 4.0 months)

ALL, acute lymphoblastic leukemia; BCMA, B-cell maturation antigen; CAR-T, chimeric antigen receptor T cell; CR, complete response; CRh, complete response with partial hematologic recovery; DLL3, delta-like canonical Notch ligand 3; DOR, duration of response; LBCL, large B-cell lymphoma; MM, multiple myeloma; ORR, objective response rate; OS, overall survival; PFS, progression-free survival; sCR, stringent complete response; SCLC, small-cell lung cancer; TCE, T cell engager; TIL, tumor-infiltrating lymphocyte.

### Oncolytic virotherapy and the rational remodeling of the microenvironment

The formidable physical and metabolic barriers constructed by solid tumors dictate that adoptive cell therapies and bispecific engagers cannot achieve curative outcomes as isolated monotherapies. The future of the field strictly relies on rational combinatorial strategies that dynamically remodel the hostile tumor microenvironment prior to or concurrent with T cell infiltration. Among the most promising of these microenvironmental modulators are oncolytic viruses. These are therapeutically engineered or naturally attenuated viral vectors, such as modified adenoviruses, herpes simplex viruses, or vaccinia viruses, designed to selectively replicate within and lyse malignant cells (116, 117). This exquisite selectivity is mechanically driven by the exploitation of defective antiviral defense pathways in cancer cells, particularly the frequent mutational inactivation of the cyclic GMP-AMP synthase and stimulator of interferon genes signaling axis, which normally severely restricts viral replication in healthy tissue (118–120).

**Figure 6:**
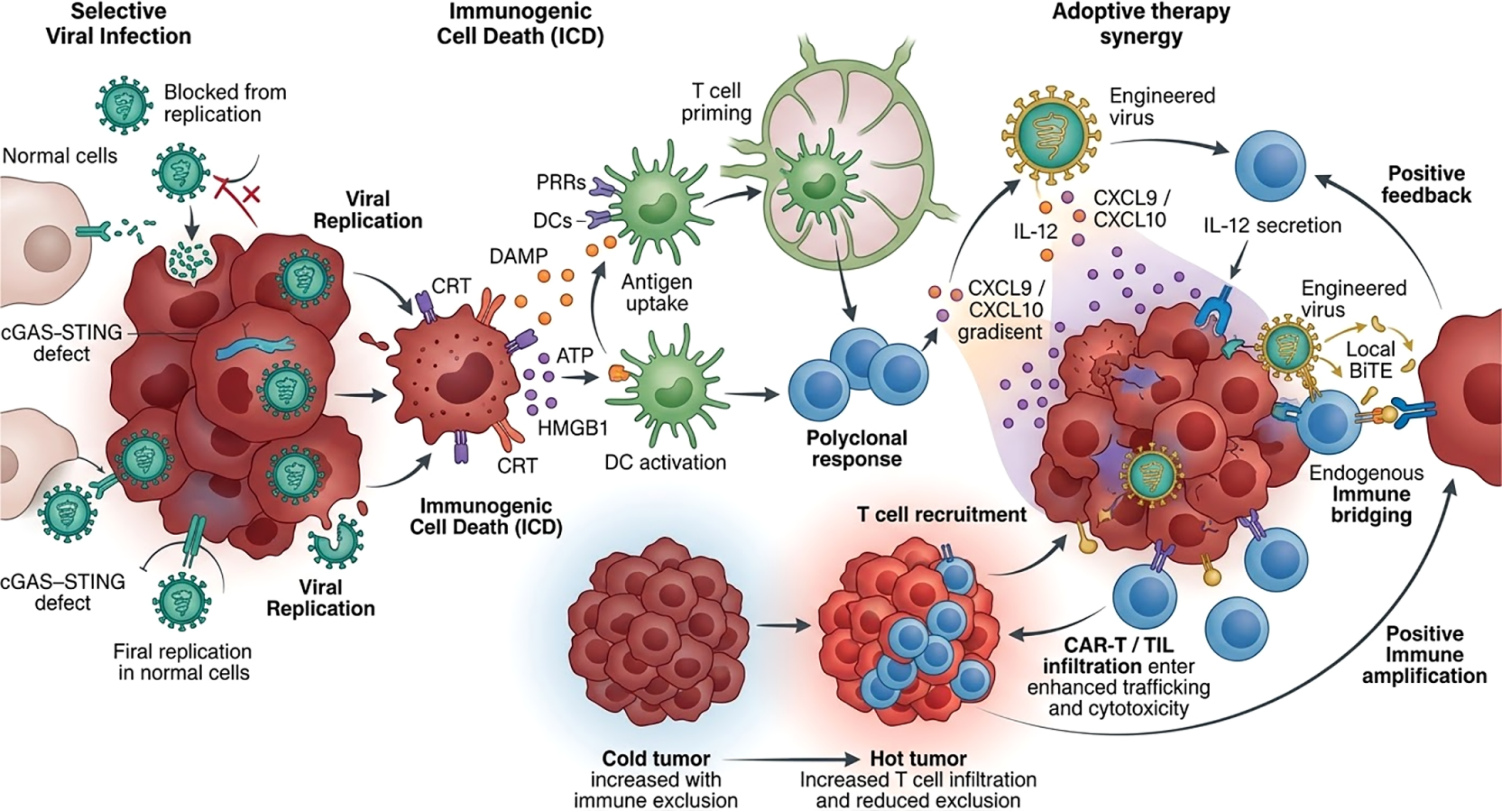
Mechanistic synergy of oncolytic virotherapy with endogenous and adoptive cellular immunotherapies. The schematic illustrates the multifaceted role of oncolytic viruses (OVs) in transforming the tumor microenvironment (TME) and amplifying anti-tumor immune responses. OVs take advantage of a cGAS–STING defect to achieve selective viral infection and replication preferentially in tumor cells, while replication is generally blocked in normal cells. The resulting intracellular viral amplification leads to tumor cell lysis, which triggers immunogenic cell death (ICD). A hallmark of ICD is the release of damage-associated molecular patterns (DAMPs), including cell-surface exposure of calreticulin (CRT) and extracellular release of ATP and HMGB1. These DAMPs are recognized by pattern recognition receptors (PRRs) on dendritic cells (DCs), initiating antigen uptake and DC activation. Upon migration, these activated DCs facilitate T cell priming, expanding the polyclonal response of endogenous T cells. This adaptive immune cascade is crucial for the visual transformation from a cold tumor, marked by high immune exclusion, into a hot tumor, characterized by increased T cell infiltration and reduced exclusion. Advanced engineering of OVs further drives adoptive therapy synergy through two primary routes of intratumoral payload delivery. First, engineered viruses secrete cytokines such as IL-12 and chemokines like CXCL9/CXCL10, creating gradients that drive T cell recruitment of both endogenous populations and adoptively transferred cells, such as CAR-T and TIL infiltration, leading to enhanced trafficking and cytotoxicity. Second, OVs can act as platforms for the local production of therapeutic molecules, such as a Local BiTE (bispecific T-cell engager). The BiTE molecules bridge Endogenous T cells directly with tumor antigens (Endogenous Immune bridging) to mediate direct killing. The culmination of these mechanisms establishes positive feedback and robust positive immune amplification loops, ultimately leading to enhanced tumor eradication.

Beyond direct cytolysis, the profound synergistic value of oncolytic virotherapy lies in its capacity to act as an immunological ignition switch, fundamentally converting an immunologically cold, stroma-rich tumor into a highly inflamed, T cell-permissive bed (121). The process of viral oncolysis induces a highly inflammatory form of programmed cell death known as immunogenic cell death. This mechanical rupture of the malignant cell forcefully expels a massive payload of previously sequestered tumor neoantigens alongside potent danger-associated molecular patterns, including extracellular adenosine triphosphate, high mobility group box 1, and surface-exposed calreticulin (122). These danger signals bind directly to pattern recognition receptors on resident dendritic cells, reversing their tumor-induced tolerogenic state and driving their maturation and migration to draining lymph nodes to prime a massive endogenous polyclonal T cell response. Furthermore, modern oncolytic vectors are aggressively engineered as localized transgene delivery vehicles. By encoding pleiotropic cytokines like interleukin-12 or critical T cell-attracting chemokines such as CXCL9 and CXCL10 directly into the viral genome, the infected tumor essentially becomes a bioreactor that actively recruits exogenously administered chimeric antigen receptor T cells or tumor-infiltrating lymphocytes deep into the tumor parenchyma (123). Even more radically, cutting-edge virotherapy constructs are being designed to secrete functional bispecific T cell engagers directly within the tumor interstitium following viral replication, thereby bridging the physical gap between highly localized viral therapy and the redirection of the systemic T cell repertoire, creating a self-amplifying loop of localized tumor eradication without the systemic toxicity typically associated with intravenous engager administration (124) (**Figure 6**).

### Next-generation synthetic immunology and epigenetic reprogramming

As T cells successfully infiltrate the tumor bed, they must be fundamentally protected against the profound immunosuppressive paracrine signals and chronic antigen stimulation that inevitably drive terminal exhaustion. This necessitates a transition from conventional singular genetic modifications to highly sophisticated synthetic gene circuits and multiplexed epigenetic reprogramming. To overcome the critical challenge of antigen heterogeneity and fatal on-target off-tumor toxicity, the vanguard of synthetic immunology is deploying Boolean logic gating to enforce absolute spatiotemporal control over T cell activation (125). The most advanced iteration of this is the synthetic Notch, or synNotch, receptor cascade (126). In an AND-gate configuration, a T cell is engineered with a synNotch receptor specific for a widely expressed tissue antigen, which does not trigger direct cytotoxicity. Upon binding this primary antigen, the synNotch receptor undergoes an intramembrane proteolytic cleavage, releasing an orthogonal transcription factor that translocates to the nucleus and actively drives the expression of a highly potent, cytolytic chimeric antigen receptor directed against a secondary, more restricted tumor antigen. This sequential recognition mechanism ensures that lethal cytolytic activity is exclusively unleashed only when the T cell physically encounters a target expressing both antigens simultaneously, thereby granting unprecedented precision in dismantling heterogeneous solid tumors while completely sparing single-antigen-positive vital organs (127). Translating synthetic biology into clinical candidates, SENTI-202 represents a first-in-class logic-gated CAR-NK cell therapy. It utilizes an ‘OR-gate’ (targeting CD33 and FLT3) combined with a ‘NOT-gate’ (protecting healthy hematopoietic stem cells via endomucin), specifically designed to treat acute myeloid leukemia (AML) while minimizing off-tumor toxicity (128). Furthermore, synNotch-gated CAR-T circuits, such as those targeting the GD2-B7H3 combination, are progressing toward Phase I trials, providing a blueprint for achieving high-precision targeting in metastatic neuroblastoma (129).

Concurrently, researchers are equipping T cells with synthetic armor designed to subvert the suppressive tumor microenvironment from within. Chimeric switch receptors represent a brilliant bioengineering manipulation of tumor-derived inhibitory signals. By physically fusing the extracellular binding domain of an inhibitory receptor, such as programmed cell death protein 1, to the potent intracellular signaling domain of a costimulatory molecule, such as CD28, engineers have created a receptor that fundamentally reverses the immunological polarity of the tumor microenvironment (130). When these armored T cells encounter PD-L1, instead of receiving a paralyzing inhibitory signal, they receive a massive burst of costimulatory activation, effectively transforming the tumor’s primary defense mechanism into a lethal vulnerability. The strategy of ‘armoring’ cells against the hostile TME has moved into late-stage clinical evaluation. BNT211 (NCT04503278) integrates a CLDN6-targeted CAR-T cell with a CAR-amplifying RNA vaccine (CAR-VAC), resulting in sustained T-cell expansion and a reported ORR of 33% in patients with advanced solid tumors, including germ cell tumors (131). Additionally, ‘switch receptor’ technologies, exemplified by BPX-601 (NCT02744287)—a rimiducid-inducible ‘GoCAR-T’ targeting PSCA—have demonstrated the ability to modulate T-cell costimulation *in vivo*, offering a controllable mechanism to enhance potency while mitigating cytokine release syndrome in pancreatic and prostate cancers (132).

**Figure 7:**
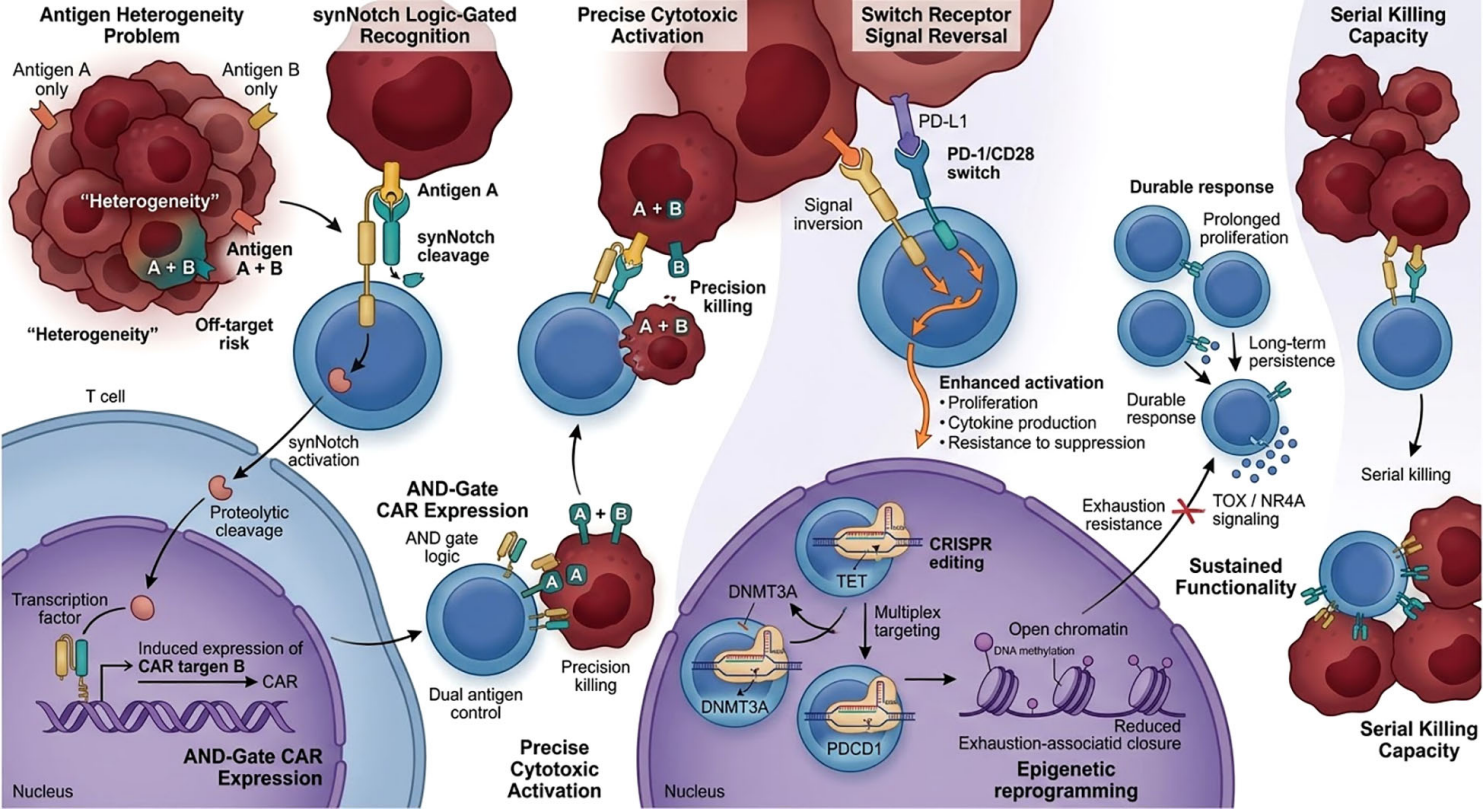
Sophisticated genetic and epigenetic engineering strategies designed to address the multifaceted challenges of treating solid tumors with CAR T cells, ranging from target recognition to persistence in the immunosuppressive tumor microenvironment. Addressing Tumor Heterogeneity for Precise Targeting. The entire engineering process is initiated in response to ‘The Antigen Heterogeneity Problem’, which remains a fundamental obstacle in solid tumor therapy. As shown in the figure, a tumor mass is not uniform but is composed of distinct subclones expressing different combinations of ‘Antigen A,’ ‘Antigen B,’ and ‘Antigen A+B.’ Conventionally targeting a single antigen creates a significant off-target risk, as single-antigen CAR T cells may kill normal healthy tissues that also express that antigen. To overcome this, intricate genetic logic circuits are engineered into T cells for dual antigen control. The central module, ‘synNotch Logic-Gated Recognition’, utilizes a synthetic Notch (synNotch) receptor system. A first receptor on the T cell binds specifically to Antigen A on a tumor cell. This binding trigger localized proteolytic cleavage and the subsequent release of a membrane-anchored transcription factor. This released transcription factor then translocates to the T cell’s nucleus, where it directly drives the induced expression of a CAR targeting Antigen B. This elegant ‘AND-gate logic’ circuit ensures that the second, activating CAR is only expressed when the primary target, Antigen A, is present, providing ‘Precise Cytotoxic Activation’. The T cell is then activated only by tumor cells expressing both ‘Antigen A+B’, leading to highly ‘Precision killing’ of double-positive tumor cells while sparing cells that express only one of the antigens, thereby minimizing off-tumor toxicity. Overcoming Immunosuppression and Preventing Exhaustion. Beyond precise recognition, T cells must navigate and overcome the tumor’s defense mechanisms. One powerful approach is ‘Switch Receptor Signal Reversal’, which utilizes a chimeric PD-1/CD28 switch receptor. By fusing the PD-1 extracellular inhibitory receptor domain to the CD28 co-stimulatory domain, the inhibitory signal from tumor-cell PD-L1 binding is not only blocked but actively converted (signal inversion) into a co-stimulatory signal. This results in enhanced activation with increased proliferation, cytokine production, and enhanced resistance to suppression within the TME, effectively turning a tumor’s shield into anactivating co-stimulatory cue. Finally, to address the long-term limitation of T cell exhaustion, the diagram illustrates ‘Epigenetic Reprogramming’. Within the T cell nucleus, multiplex targeting via CRISPR-Cas9 is used to knock out or modify key epigenetic regulators, such as DNA methyltransferase DNMT3A and methylcytosine dioxygenase TET, as well as inhibitory receptors like PDCD1 (encoding PD-1). This fundamental epigenetic reprogramming prevents ‘exhaustion-associated closure’ and maintains a functionally active ‘open chromatin’ state. This open chromatin effectively blocks key exhaustion-driving signaling pathways, such as TOX/NR4A signaling, leading to ‘Exhaustion resistance’ and ensuring ‘Sustained Functionality’ The collective outcomes of these engineering strategies are visualized in the far right of the figure, demonstrating ‘Sustained Functionality’ and a dramatic ‘Serial Killing Capacity’. These exhaustion-resistant T cells exhibit a ‘durable response’ with ‘prolonged proliferation’ and ‘long-term persistence’ within the tumor, allowing a single T cell to continuously and sequentially eliminate multiple tumor cells, achieving multi-round ‘serial killing’ without becoming functionally exhausted, ultimately driving more durable antitumor responses.

Furthermore, the advent of highly efficient CRISPR-Cas9 ribonucleoprotein electroporation has unlocked the ability to permanently reprogram the epigenetic fate of therapeutic T cells prior to infusion. Beyond simple programmed cell death protein 1 knockouts, contemporary multiplex genome editing targets master regulatory enzymes governing the epigenetic landscape of exhaustion. The targeted disruption of critical DNA methyltransferases, such as DNMT3A, or members of the TET family of demethylases, fundamentally prevents the chromatin closure and DNA methylation that lock T cells into the terminally exhausted state (133). By physically eliminating the genetic loci responsible for translating chronic stimulation into functional anergy, these hyper-engineered, epigenetically youthful T cells can sustain relentless, high-avidity serial killing deep within the metabolic desert of the solid tumor for months or even years (134) (**Figure 7**).

### Decentralized bioprocessing and the allogeneic off-the-shelf frontier

The ultimate realization of T cell immunotherapy as a foundational pillar of global oncology cannot rely on the current bespoke, artisan model of autologous biomanufacturing. The logistical fragility, astronomical production costs, and critical weeks-long manufacturing delays inherent to autologous chimeric antigen receptor and tumor-infiltrating lymphocyte therapies fundamentally restrict patient access and frequently result in disease progression before the living drug can be administered. The absolute prerequisite for democratizing these advanced therapeutics is a definitive paradigm shift toward highly automated, decentralized, closed-system bioprocessing, ultimately culminating in the development of universal, allogeneic off-the-shelf cellular products. Transitioning from patient-derived lymphocytes to healthy donor-derived apheresis products, or infinitely expandable induced pluripotent stem cell lines, immediately resolves the issues of variable cellular fitness and protracted manufacturing timelines, generating a universally available therapeutic inventory that functions pharmacologically akin to bispecific T cell engagers (135).

However, the deployment of allogeneic T cells introduces two massive immunological barriers that require the absolute pinnacle of multiplexed genomic engineering: graft-versus-host disease and host-mediated allograft rejection. To prevent the infused donor T cells from recognizing the recipient’s tissues as foreign and triggering fatal graft-versus-host disease, the endogenous T cell receptor alpha constant locus must be completely and cleanly disrupted utilizing targeted nucleases like CRISPR-Cas9 or transcription activator-like effector nucleases (136). The transition toward allogeneic, ‘off-the-shelf’ platforms is epitomized by ALLO-501A (NCT04416984), a TALEN-edited anti-CD19 CAR-T cell product. Early-phase data from the ALPHA2 study demonstrated a manageable safety profile and substantial efficacy in relapsed/refractory large B-cell lymphoma, mirroring the responses seen in autologous products (137). Similarly, CYAD-101 (NCT03692429), which utilizes a non-gene-edited approach via a T-cell receptor inhibitory molecule (TIM), has shown promising preliminary stability and safety in the treatment of refractory metastatic colorectal cancer, confirming the viability of allogeneic T-cell engagement in solid tumor indications (138). Simultaneously, to prevent the patient’s intact endogenous immune system from immediately rejecting the therapeutic allograft, the synthetic cells must be rendered immunologically invisible. This stealth phenotype is engineered by knocking out the beta-2 microglobulin gene, which completely abolishes the surface expression of all major histocompatibility complex class I molecules, effectively hiding the cells from the host’s cytotoxic CD8 positive T cell compartment (139).

Yet, this engineered loss of major histocompatibility complex class I triggers a secondary evolutionary defense mechanism: the missing-self recognition pathway of host natural killer cells, which are primed to rapidly eliminate any cell lacking classical human leukocyte antigen expression. To circumvent this natural killer cell-mediated destruction, cutting-edge allogeneic platforms are executing highly complex knock-in strategies, utilizing adeno-associated viral vectors to precisely insert genes encoding non-classical, immunosuppressive human leukocyte antigen molecules, such as human leukocyte antigen E or human leukocyte antigen G, directly into the disrupted beta-2 microglobulin locus (130). This sophisticated molecular camouflage perfectly mimics the immune evasion strategies of the maternal-fetal interface, generating a universally compatible, universally lethal population of synthetic effector cells (140). The convergence of these advanced allogeneic platforms with highly specific, multiplexed synthetic receptors and virally remodeled tumor microenvironments represents the absolute zenith of modern immunotherapy. It signals a near-future landscape where the devastating complexity of the solid tumor is finally matched and overwhelmed by the boundless architectural flexibility of engineered immunity.

## Conclusion

The era of T cell-driven immunotherapy has unequivocally redefined the oncological landscape, validating the cytotoxic T lymphocyte as the most potent entity capable of durable cancer eradication. As detailed in this review, the clinical ascendance of chimeric antigen receptor T cells, tumor-infiltrating lymphocytes, and bispecific T cell engagers represents the triumphant convergence of synthetic biology, endogenous immune surveillance, and advanced protein engineering. Each modality addresses the fundamental challenge of tumor recognition through a distinct mechanistic lens: chimeric antigen receptors impose potent, major histocompatibility complex-independent synthetic specificity; tumor-infiltrating lymphocytes harness the natural, highly multiplexed polyclonal repertoire required to counteract mutational burden; and T cell engagers bypass *ex vivo* cellular manipulation to rapidly redirect systemic immunity via pharmacologic bridging. Yet, the stark dichotomy between the curative successes in hematologic malignancies and the widespread recalcitrance of solid tumors underscores the profound limitations of deploying any single modality against a dynamically evolving, heterogeneous disease.

The ultimate realization of broad, curative immunotherapy dictates a fundamental paradigm shift away from iterative monotherapies toward rationally designed, combinatorial counter-offensives. The solid tumor microenvironment—a formidable fortress characterized by profound metabolic starvation, dense physical exclusion, and active cellular suppression—demands therapeutic strategies that are intrinsically multimodal. As we move forward, the absolute frontier of oncology lies in the seamless integration of these cellular and pharmacological pillars with transformative microenvironmental modulators, such as oncolytic viruses, and the relentless advancement of multiplexed genetic engineering. By equipping therapeutic T cells with logic-gated synthetic circuits to safely navigate antigen heterogeneity, deploying precise CRISPR-mediated epigenetic reprogramming to permanently resist terminal exhaustion, and establishing robust, universally compatible allogeneic biomanufacturing platforms, we can systematically dismantle the tumor’s evolutionary defenses. Ultimately, the future of cancer eradication will not rely on a singular immunological magic bullet, but on the orchestrated, synergistic deployment of the entire T cell armamentarium, dynamically tailored to the precise spatiotemporal complexity of both the malignancy and the host immune system.
